# The architecture of salt tolerance: A multi-scale view of sodium transport in plants

**DOI:** 10.1016/j.xplc.2026.101888

**Published:** 2026-05-12

**Authors:** Víctor J. Fernández-Ramírez, Alfonso G. De la Rubia, Jose M. Pardo, Francisco J. Quintero, Francisco M. Gámez-Arjona

**Affiliations:** 1 Institute of Plant Biochemistry and Photosynthesis, Spanish National Research Council (CSIC) – University of Seville, 41092 Seville, Spain

**Keywords:** ion transporters, sodium, single-cell analysis, nutrient translocation regulation, saline plant tolerance

## Abstract

Soil salinity compromises agricultural productivity by disrupting plant ionic and osmotic equilibrium. While sodium (Na^+^) is the principal cytotoxic ion in saline soils, it can also function as a beneficial element at low concentrations, contributing to cellular osmotic balance and partially offsetting potassium (K^+^) requirements for turgor. Resolving how plants manage this concentration-dependent duality is critical for developing salt-tolerant crops. Achieving this objective requires a comprehensive understanding of how plants regulate Na^+^ via mechanisms such as ion sensing, membrane transport, subcellular sequestration, and tissue-level distribution. Such insights are critical for developing crop varieties with enhanced salt tolerance without compromising yield. To address this fundamental topic, this review integrates data from classical approaches and single-cell RNA sequencing (scRNA-seq) analyses to provide a multi-scale view of Na^+^ homeostasis. We leverage publicly available scRNA-seq datasets to generate cell-type-specific expression profiles of the primary Na^+^ transporter SOS1 and its Ca^2+^-dependent regulators, as well as high-affinity K^+^ transporters (HKTs) that contribute to Na^+^/K^+^ balance. This approach reveals key differences in how dicotyledons (*Arabidopsis thaliana*) and monocotyledons (*Oryza sativa*) regulate ion levels. This review also highlights the significance of subcellular localization and endomembrane trafficking of ion transporters, which determine transporter density and stability at target membranes. By connecting cellular-level mechanisms to tissue-level organization, our synthesis tackles a pressing question in agriculture and biology: how do plants coordinate ion movement across different spatial domains to survive in saline environments? This integrated perspective offers mechanistic insights into plant salinity tolerance and supports the development of salt-resilient crops.

## Teaser

Plant salinity tolerance depends on how cells regulate sodium and potassium homeostasis through specific ion transporters. This review summarizes current insights into salinity-related ion transport, including signal perception, ion translocation, and intracellular compartmentalization, supported by cell-type-specific expression profiles and comparative analyses between dicotyledonous and monocotyledonous model species.

## Beyond toxicity: The intricate interplay between sodium and potassium in plant systems

**Figure 1:**
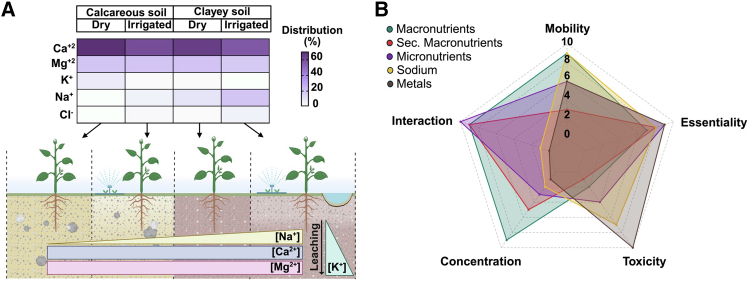
Comparative analysis of mineral properties in plant tissues and the effects of soil composition and anthropogenic activities on ion levels. **(A)** Relative distribution of major exchangeable ions, including Na^+^, K^+^, Ca^2+^, Mg^2+,^ and Cl^−^, by soil type (calcareous vs. clayey soils) and agricultural management system (irrigated vs. rainfed), based on a synthesis of published data (Abbaslou et al., 2020; Bolan et al., 2023; Baki et al., 2025; Hui et al., 2025). The heatmap shows increased Na^+^ concentration in clayey soils, especially under irrigation, while Mg^2+^ and Cl^−^ levels remain constant. K^+^ leaching further exacerbates Na^+^ accumulation in irrigated clayey soils. **(B)** Radar plot comparing macronutrients (green), secondary macronutrients (red), micronutrients (purple), Na^+^ (yellow), and metals (brown) across five parameters: mobility, essentiality, toxicity, plant concentration, and interactions with other ions. Each shaded region represents the relative score on a 0–10 scale for each ion group in each category. The plot highlights the unique characteristics of Na^+^ compared with other ions. Its high mobility, strong interactions, and potential toxicity place Na^+^ more closely with toxic ions. However, its partial essentiality and accumulation in plant tissues also associate Na^+^ with nutrient ions, placing it in an intermediate position between these two categories.

Soil salinization, exacerbated by climate change and unsustainable land use, represents a growing global threat to agricultural productivity, ecosystem integrity, and food and water security (Figure 1A) (https://openknowledge.fao.org/handle/20.500.14283/cd3044en) (Van Zelm et al., 2020). Effective mitigation demands integrated strategies encompassing technological innovation, improved land-use practices, and coordinated international policy (Hirt et al., 2023). Although sodium (Na^+^) toxicity is central to the disruption caused by salinization, its effects are concentration- and species-dependent, underscoring the importance of understanding Na^+^ transport mechanisms for improving plant performance under salt stress. Low levels of Na^+^ can support leaf anatomical development, enhance carbon assimilation, and partially offset K^+^ requirements for turgor; in C_4_ species, Na^+^ is essential, and its omission produces deficiency symptoms (Brownell and Crossland, 1972; Boag and Brownell, 1979; Johnston et al., 1984; Battie-Laclau et al., 2014). However, such benefits diminish rapidly as Na^+^ concentration increases. Na^+^ possesses unique physicochemical properties that distinguish its impact on plant fitness from that of other ions; notably, the high mobility of Na^+^ in soil and the vascular system allows it to rapidly outcompete essential cations for transport and cellular uptake (Figure 1B), necessitating tight regulation in Na^+^ sensitive species. Excess Na^+^ causes osmotic stress, nutrient imbalance, oxidative damage, leaf necrosis, and growth inhibition, largely through competition with K^+^, which is essential for enzymatic activity (Ragel et al., 2019). Thus, a low cytosolic Na^+^/K^+^ ratio is crucial for plant survival in saline environments (Raddatz et al., 2020).

Plant Na^+^ uptake is highly diverse and lacks a single dominant pathway (Ma et al., 2026). Most Na^+^ entry occurs through nonselective cation channels, including salt-induced cyclic nucleotide-gated channels (CNGCs), such as *Arabidopsis thaliana* CNGC19 and CNGC20 (Kugler et al., 2009). Glutamate-like receptors have been proposed to facilitate Na^+^ influx due to their limited ion selectivity (Davenport, 2002); however, substantive genetic evidence is still lacking; their involvement in salinity tolerance may be restricted to Ca^2+^ signaling (Simon et al., 2023). Similarly, certain aquaporins, such as PIP2;1 and PIP2;2 in *A. thaliana* and PIP2;4 in rice, have also been proposed as Na^+^ transporters, although direct *in planta* experimental evidence is lacking (Tran et al., 2025). Additionally, the nitrate transporters AtNRT1.1/NPF6.3 and AtNRT1.2/NPF4.6 may mediate Na^+^ entry, as saline conditions allow Na^+^ to substitute for H^+^ during proton-coupled nitrate symport (Álvarez-Aragón and Rodríguez-Navarro, 2017; Liu et al., 2025b). Although high-affinity transporters, such as members of the KUP/HAK/KT family, may contribute to Na^+^ movement under specific nutrient conditions, their predominantly internal expression suggests that they are unlikely to serve as primary routes for Na^+^ uptake (Demidchik and Maathuis, 2007; Horie et al., 2007; Zhang et al., 2010; Wang et al., 2024a). Transient Na^+^ influx can aid osmotic adjustment during sudden salt shock, acting as a “cheap” osmolyte until exclusion mechanisms are activated. Moreover, multiple symplastic transport pathways operate alongside apoplastic entry, which can reach the xylem where endodermal barriers are weakened, such as at sites of lateral root emergence or in the meristem (Franke, 2015; Cui et al., 2021; Cantó-Pastor et al., 2025).

**Figure 2:**
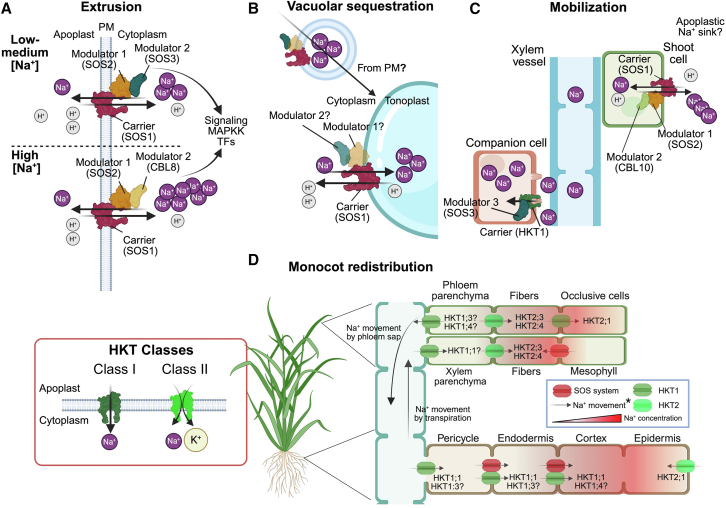
Detoxification mechanisms for Na^+^ in plants. **(A–C)** Extrusion, in which Na^+^ is exported from the cytoplasm to the apoplast by SOS1, regulated by SOS2 and SOS3 **(A)**; vacuolar sequestration, in which Na^+^ is stored in vacuoles via tonoplast transporters, also modulated by SOS proteins **(B);** and mobilization, in which Na^+^ is redistributed from the xylem to companion cells and aerial tissues using HKT1 carriers **(C)**. **(D)** Monocot redistribution, in which grasses use a large set of HKT proteins, comprising two classes: class I, which mediates Na^+^ uptake into the cell, and class II, which mediates coupled Na^+^ and K^+^ transport. In this model, Na^+^ uptake is promoted by HKT2;1 in the root, whereas the SOS system is localized in the inner layers of this organ. Some aerial tissues, such as fibers and guard cells, are also enriched in Na^+^. The arrows illustrate Na^+^ symplastic flow; apoplastic flow pathways are not depicted.

How plants sense sodicity stress at the cellular and whole-plant levels remains uncertain. Glycosyl inositol phosphorylceramide sphingolipids are the only identified candidate Na^+^ sensors on the root surface in plants (Jiang et al., 2019), but the involvement of this mechanism in downstream whole-plant salt tolerance is not fully understood. Upon salt perception, Na^+^ stress triggers Ca^2+^ waves that propagate through tissues and are decoded by a network of calcineurin B-like proteins (CBLs), calcium-dependent protein kinases, calmodulins, and calmodulin-like proteins, converting ionic signals into targeted stress responses (Dodd et al., 2010; Kudla et al., 2010; Reddy et al., 2011; Steinhorst et al., 2022). Central to salt tolerance is the Ca^2+^-dependent salt overly sensitive (SOS) pathway (Figure 2) (Ali et al., 2023; Ma et al., 2026). The plasma-membrane Na^+^/H^+^ antiporter SOS1 expels Na^+^ using the proton gradient and is activated by the kinases SOS2 (CIPK24) and CIPK8, which in turn depend on Ca^2+^-bound CBLs such as CBL4 (SOS3), CBL8, and CBL10. Beyond their primary roles in Na^+^ exclusion, SOS1 modulators coordinate other pathways. SOS2 serves as a versatile regulator of diverse targets, including vacuolar H^+^-ATPases (Batelli et al., 2007), the cation exchanger CAX1 (Cheng et al., 2004), K^+^ channels such as AKT1 (Li et al., 2023), and nitrate transporters such as NRT1.2 (Liu et al., 2025b), thereby helping to maintain overall mineral homeostasis and avoid uncontrolled Na^+^ influx. CBL isoforms act as spatial cues that recruit SOS2 to specific membranes to fine-tune ion and nutrient transport across tissues (Ma et al., 2026). Complementing SOS-driven Na^+^ exclusion, the high-affinity K^+^ transporter (HKT) family governs Na^+^ and K^+^ distribution at multiple scales (Figure 2) (Hamamoto et al., 2015; Ali et al., 2021; Li et al., 2025). Class I HKTs, present in both monocotyledons and dicotyledons, primarily retrieve Na^+^ from the apoplastic space into xylem parenchyma cells to protect leaves and reproductive organs. Class II HKTs, found only in monocotyledons, transport both Na^+^ and K^+^, enabling Na^+^ uptake during K^+^ starvation to partially fulfill the role of K^+^ in maintaining osmotic balance (Horie et al., 2007). Overall, Na^+^ tolerance relies on a multilayered network that integrates sensing, signaling, and coordinated transporter activity. Deciphering this system is essential for developing salt-tolerant crops adapted to future climates.

## Anatomical and physiological responses to Na^+^ stress

### Root architecture and Na^+^ homeostasis

To understand how plant transporters respond to and regulate Na^+^ levels, we must first examine the root architecture that defines the first physical barriers and pathways these ions must navigate. Na^+^ homeostasis in roots must be understood in the context of both longitudinal and radial tissue architecture. Along the longitudinal axis, a developmental gradient spans from the apical meristem through zones of cell division, elongation, and differentiation, ultimately leading to specialized, position-dependent cell fates (Schiefelbein and Benfey, 1991; Somssich et al., 2016). These differentiation processes are responsive to environmental cues, including salinity (Zou et al., 2022). The functional specialization of different regions of the root is reflected in the receptor-like kinase SCHENGEN3/GASSHO1 (GSO1), which protects distinct longitudinal zones of the root through interactions with region-specific partners (Chen et al., 2023). In the meristem, GSO1 stimulates the SOS2–SOS1 complex to promote Na^+^ efflux, preserving root growth in a region with high ion permeability. Although this permeability supports root growth, it also renders the meristem a major entry point for Na^+^, potentially compromising cellular function and development under saline conditions (Liu et al., 2015; Byrt et al., 2018). This reflects a structural trade-off between growth plasticity and ion exclusion. Conversely, in mature root regions, the Casparian strip (CS) reduces ion permeability and elicits distinct regulatory responses: salt stress induces the accumulation of GSO1 in the endodermis, where it binds CS integrity factor peptides to reinforce the CS integrity factor–GSO1–SCHENGEN1 barrier, thereby restricting Na^+^ influx (Chen et al., 2023). Similarly, epidermal cells specialize into trichoblasts and atrichoblasts in a radial pattern governed by hormone crosstalk (Vissenberg et al., 2020). Apoplastic pH, which is critical for cell expansion, ion transporter activity, and stress responses (Gámez-Arjona et al., 2022), shows a pronounced radial gradient in *A. thaliana* roots (Martinière et al., 2018), probably supporting ion transport directionality. The endodermis and stele have more acidic apoplastic conditions than outer cell layers. This radial pH gradient is shaped by the CS, cell wall properties, proton pumps, and Ca^2+^ signaling. Together with longitudinal tissue zonation, this spatial organization governs ion flux between the soil and vasculature, underpinning Na^+^ homeostasis and stress resilience. Once Na^+^ reaches the shoot, its impact on plant fitness depends largely on its spatial distribution within the leaf. The following discussion of leaf architecture provides the structural framework needed for understanding how specific transporters sequester Na^+^ away from salt-sensitive photosynthetic tissues.

### Leaf architecture and Na^+^ homeostasis

Leaves comprise specialized tissues that coordinate photosynthesis and ion homeostasis under salt stress. The epidermis, formed by pavement and guard cells, limits water loss and regulates gas exchange. Beneath this layer, the palisade mesophyll contains chloroplast-rich columnar cells for light capture, while the spongy mesophyll facilitates CO_2_ diffusion via intercellular air spaces. Vascular bundles transport water and nutrients through the xylem and distribute photosynthates and ions via the phloem. Under saline conditions, this structural framework is both an asset and a vulnerability. Salinity induces anatomical modifications, such as thickening of the epidermal layers and cuticular waxes and increased palisade mesophyll density, which enhance water retention and support photosynthetic efficiency despite osmotic stress (Longstreth and Nobel, 1979; Shepherd and Griffiths, 2006; Munns and Tester, 2008). However, Na^+^ accumulation in mesophyll cells via the transpiration stream and apoplast disrupts cytosolic Na^+^/K^+^ balance, impairing metabolism and photosynthetic efficiency (Masarmi et al., 2023).

To minimize further Na^+^ uptake, stomata partially close via an abscisic acid (ABA)-mediated signaling cascade that involves reactive oxygen species, nitric oxide (NO), and Ca^2+^ as secondary messengers (Arif et al., 2020; Hsu et al., 2021; Bharath et al., 2021). The capacity to sustain growth under salinity hinges on leaf-level adaptations that optimize the trade-off between photosynthetic activity and salt-stress mitigation. In tolerant species, this optimization is achieved through a suite of integrated mechanisms, including refined stomatal control, enhanced ion exclusion (e.g., at the leaf sheath or petiole), and efficient vacuolar sequestration of Na^+^ (Shabala, 2013). Ultimately, these foliar strategies complement root-based ion exclusion and transport, contributing synergistically to whole-plant ionic homeostasis and stress resilience. The effectiveness of these architectural defenses depends on the plant’s capacity to regulate ion movement between organs. To understand how plants direct Na^+^ either toward or away from specific anatomical sites, it is essential to examine the molecular mechanisms controlling long-distance Na^+^ transport and partitioning.

## Long-distance Na^+^ transport and partitioning

Salt tolerance in plants relies on systemic responses, with long-distance ion transport and transpiration playing central roles (Munns and Tester, 2008; Wu, 2018; Atta et al., 2023). High transpiration can lead to toxic accumulation of ions such as Na^+^, chloride (Cl^−^), and heavy metals in shoots, while low transpiration may cause deficiencies of phloem-immobile nutrients such as Ca^2+^ and boron, particularly in developing tissues (Montanaro et al., 2015; Chatzistathis et al., 2021). In *Chenopodium quinoa,* 200 mM NaCl reduced transpiration by approximately 60% compared with control conditions (Jaramillo Roman et al., 2021). In wheat, salt stress caused substantial decreases in both daytime and nighttime transpiration rates, primarily by reducing stomatal conductance (Nguyen and Stangoulis, 2024). This acclimation response helps minimize water loss under high-salt stress (Jaramillo Roman et al., 2021; Sharmin et al., 2021). ABA-induced stomatal closure reduces root-to-shoot xylem water flow, thereby limiting the total amount of salt ions transported to leaves (Munns and Tester, 2008; Fricke, 2020; Hsu et al., 2021).

Despite reduced transpiration, plants can maintain ion flux to shoots via mechanisms such as root pressure (Jing et al., 2025). Under salinity, water transport shifts from apoplastic to transmembrane pathways. Xylem-associated parenchyma cells regulate water relations and facilitate stress recovery, including embolism repair (Secchi and Zwieniecki, 2016). These living cells, strategically positioned adjacent to xylem conduits, sustain hydraulic regulation and water transport efficiency (Scoffoni et al., 2017). Interactions among transpiration, ABA signaling, and osmotic adjustment, together with the activity of ion transporters in specific cells, underscore the complexity of plant responses to salinity.

The activities of Na^+^-permeable transporters (HKT1-like proteins) and Na^+^/H^+^ antiporters (e.g., SOS1) in xylem parenchyma cells contribute significantly to the regulation of Na^+^ loading into the transpiration stream (Oh et al., 2009; Hamamoto et al., 2015; El Mahi et al., 2019; Gámez-Arjona et al., 2024). Studies of wheat cultivars with differential salt tolerance revealed that salt-resistant varieties exhibited enhanced expression of HKT1;5-like transporters in xylem parenchyma cells, facilitating Na^+^ unloading from xylem sap before it reaches photosynthetic tissues (Byrt et al., 2007). In barley, a selective increase in K^+^ loading over Na^+^ by xylem parenchyma cells preserves favorable Na^+^/K^+^ ratios and hydraulic conductivity (Houston et al., 2020). Moreover, inverse regulation of SOS1 and HKT1 stability, depending on the intensity of sodicity stress, coordinates long-distance Na^+^ transport in *A. thaliana* (Gámez-Arjona et al., 2024). HKT1 also mediates Na^+^ recirculation via phloem loading (Berthomieu et al., 2003), and *SOS1* transcription is diurnally regulated to match daily evapotranspiration cycles (Cha et al., 2022).

## Cell-type-specific expression of Na^+^ transporters: A spatial framework for Na^+^ homeostasis and salt tolerance

To address current gaps in our understanding of Na^+^ homeostasis, we analyzed publicly available single-cell RNA sequencing (scRNA-seq) datasets from both dicotyledons and monocotyledons. Specifically, we examined root and leaf data from *A. thaliana* and *Oryza sativa*, obtained from the Plant scRNA-Seq Browser and the Rice Multi-Omics Atlas (Denyer et al., 2019; Ma et al., 2020; Kim et al., 2021; Wang et al., 2026, 2025). See supplemental data for further details.

### Single-cell analysis in dicotyledons

**Figure 3:**
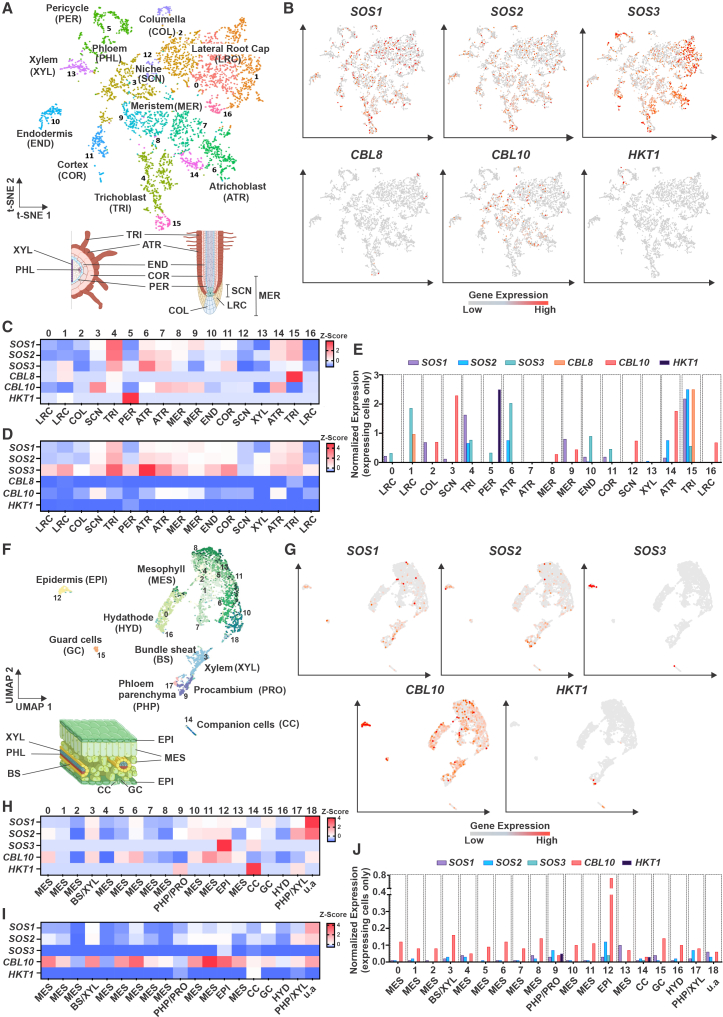
Single-cell analysis of Na^+^ homeostasis components in *A. thaliana*. **(A)** Two-dimensional t-SNE (t-distributed stochastic neighbor embedding) projection of 4,727 single-cell transcriptomes from *A. thaliana* roots, grouped into 17 transcriptionally distinct clusters corresponding to major root cell types, as defined in the Arabidopsis Root scRNA-Seq Atlas (Denyer et al., 2019; Ma et al., 2020). **(B)** Individual t-SNE plots showing the spatial expression patterns of *SOS1*, *SOS2*, *SOS3*, *CBL8*, *CBL10*, and *HKT1* across *A. thaliana* root cell clusters. Expression intensity reflects transcript abundance within each cell, revealing distinct enrichment patterns across specific cell types. **(C)** Heatmap showing the proportion of cells expressing each gene within each cluster. Values are row-normalized to highlight relative expression prevalence across cell types. **(D)** Heatmap showing the proportion of cells expressing each gene across clusters. Values are globally normalized across all genes and clusters, allowing direct comparison of absolute expression magnitudes. **(E)** Bar plot showing the average expression level of each gene across cell-type clusters. **(F)** Two-dimensional UMAP (uniform manifold approximation and projection) of 5,230 single-cell transcriptomes from *A. thaliana* leaves, grouped into 19 transcriptionally defined clusters corresponding to major leaf cell types, as reported by Kim et al. (2021). **(G)** Individual UMAP plots showing the spatial expression patterns of *SOS1*, *SOS2*, *SOS3*, *CBL10*, and *HKT1* across *A. thaliana* leaf cell clusters. *CBL8* is excluded due to undetectable expression levels in shoot-derived cells. **(H–J)** Heatmaps **(H and I)** and bar plot **(J) corresponding** to **(C)**, **(D)**, and **(E)**, respectively, depicting the proportion of cells expressing each gene across leaf clusters. Cluster 18 (unassigned [u.a.]) lies close to the mesophyll cluster but has a specific gene expression signature that differs from that of mesophyll cells; therefore, it is indicated as an unassigned cluster.

A detailed visualization of the scRNA-seq analysis of *A. thaliana* roots is shown in Figures 3A–3E. *SOS1* expression was detected across several root cell types (Figures 3B and 3C), with pronounced enrichment in epidermal cells, particularly trichoblasts, as well as in the meristem and cortex. A similar expression pattern was observed for *SOS2* and *SOS3* (Figures 3B and 3C), with both genes showing peak expression levels in epidermal cell types, notably trichoblasts and atrichoblasts, mirroring the expression profile of *SOS1*. *SOS3* exhibited the highest overall transcript abundance among the three genes (Figures 3D and 3E). However, the distribution of SOS3 transcripts should be interpreted with caution because protoplasting-induced Ca^2+^ influx during cell isolation can artificially activate calcium-responsive pathways, potentially leading to non-physiological induction of *SOS3* (Denyer and Timmermans, 2022). The co-enrichment of *SOS1*, *SOS2*, and *SOS3* in these regions may indicate a key role for the SOS pathway in mediating Na^+^ efflux at the root–soil interface while simultaneously protecting the quiescent center from ionic fluctuations. The presence of SOS components in trichoblasts is particularly notable, given their critical role as specialized environmental sensors in *A. thaliana* roots that integrate multiple stress-sensing mechanisms (Ibeas et al., 2024). Although the core SOS components are well studied, the roles of other salt-tolerance regulators, such as CBL8 and CBL10, especially their tissue-specific functions, remain unclear. *CBL8* was detected in trichoblasts (Figure 3C), where it may function during chronic salt exposure (Steinhorst et al., 2022). By contrast, *CBL10* showed a distinct accumulation pattern, with enrichment at the tip of the root meristematic zone (Figure 3C), suggesting an additional role in calcium signaling, potentially controlling cell division and differentiation, apart from the previously reported role in intracellular Na^+^ sequestration and shoot-to-root signaling (Kim et al., 2007; Quan et al., 2007; de la Torre et al., 2013).

Unlike the broad epidermal expression of SOS pathway components, *HKT1* expression was restricted to the pericycle, consistent with its role in enabling selective Na^+^ retrieval from the xylem, with minimal expression in other root cell types (Figures 3B–3E). This low and spatially restricted distribution is consistent with the well-established role of HKT1 in preventing excessive Na^+^ translocation to the shoot (Sunarpi et al., 2005; Gámez-Arjona et al., 2024), rather than in mediating early salt sensing or exclusion at the root surface. Together with other SOS components, HKT1 plays a role in protecting metabolically active root tissues that are particularly vulnerable to environmental stress.

Complementing the root analysis, scRNA-seq profiling of *A. thaliana* leaf tissues revealed distinct clusters corresponding to major leaf cell types (Figure 3F) (Kim et al., 2021). *SOS1* was detected across multiple cell types, including mesophyll, epidermal, and guard cells, with lower levels observed in phloem parenchyma and bundle sheath cells (Figures 3G, 3H, and 3J). Although this broad pattern initially seems to contrast with the strong vascular expression reported by *proSOS1:GUS* (Shi et al., 2002), analysis of this dataset confirmed the presence of SOS1 transcripts in phloem parenchyma and bundle sheath cells (Figure 3J). The perceived difference in signal intensity is readily explained by the two techniques: scRNA-seq provides a temporal snapshot of transcript levels and is prone to underrepresenting lignified vascular cells. In contrast, GUS reporter assays provide cumulative readouts, allowing signals from even low-level expression to integrate over time. Thus, our analysis complements the reporter findings and reinforces the role of SOS1 in regulating long-distance Na^+^ transport. *SOS2* also displayed a widespread expression pattern, with moderate enrichment in mesophyll cells, phloem parenchyma, and xylem (Figures 3G and 3H). Both *SOS1* and *SOS2* exhibited elevated expression in a cell cluster with transcriptomic features similar to those of mesophyll cells; however, distinct molecular signatures indicate that this cluster represents an uncharacterized cell type (unassigned cluster 18; Figure 3H). The broad and coordinated expression of these two SOS components in leaves likely reflects a critical role in protecting the photosynthetic machinery from progressive ion accumulation driven by evapotranspiration and from salt-induced damage under long-term mild saline conditions. Conversely, *SOS3* was highly restricted to the epidermis, where only a small subset of cells showed detectable expression (Figures 3G and 3H). In this region, SOS3 likely contributes to ion regulation during transpiration and may also function as a stress sensor, initiating calcium-mediated signaling in response to environmental cues beyond salt stress. However, as illustrated in Figures 3I and 3J, the overall detection of SOS pathway components in leaf tissues was markedly lower than in roots, as noted above. By contrast, *CBL10* was highly expressed across all leaf tissues, surpassing the expression levels of other SOS pathway components (Figures 3I and 3J). Notably, *CBL10* showed strong expression in both epidermal and mesophyll cells (Figures 3G and 3H), in line with its well-established role in shoot-based salt tolerance (Quan et al., 2007). In contrast, *CBL8* was not detected, suggesting a more prominent role in roots (Steinhorst et al., 2022). Similar to its restricted expression in the root pericycle, *HKT1* displayed tight spatial regulation in shoots, with expression predominantly confined to phloem parenchyma and companion cells (Figures 3G and 3H). Although sparse, low-intensity *HKT1* signals could be detected in non-vascular areas such as mesophyll cells, quantitative analysis indicated that these levels fell at or below reliable detection thresholds. This pattern most likely reflects the technical background inherent to scRNA-seq rather than robust biological expression; nonetheless, a minor functional role in leaf Na^+^ homeostasis under high-salinity conditions cannot be entirely excluded. This largely vascular-restricted localization further supports a specialized role for HKT1 in vascular ion homeostasis rather than surface-level stress sensing. These data align with its proposed role in Na^+^ recirculation from shoots to roots, most likely by mediating Na^+^ loading into the phloem sap in aerial tissues and unloading in the root vasculature, and with its newly identified role in protecting reproductive organs in *A. thaliana* (Berthomieu et al., 2003; Uchiyama et al., 2023). This mechanism helps prevent cytotoxic Na^+^ accumulation in leaf tissues by facilitating long-distance transport via the phloem, rather than direct retrieval from the transpiration stream. Consistently, *HKT1* transcript levels in leaf tissues remained low, similar to those of SOS pathway components (Figures 3I and 3J), reinforcing the spatial and functional restriction of these Na^+^ transport and regulatory modules within the shoot.

### Single-cell profiling of root gene expression under salt stress

Available scRNA-seq data from salt-stressed roots were used to analyze the expression of Na^+^ homeostasis regulators, as shown in Supplemental Figure 1 (Wang et al., 2026). After a one-day treatment with 100 mM NaCl, *SOS1* displayed enhanced expression primarily in the epidermis and endodermis. This targeted upregulation in these tissue layers supports a predominant role for SOS1 in protecting cells most exposed to salt stress. Moreover, the higher expression in trichoblasts points to an additional role in early salt-stress perception. *SOS2* exhibited a spatial expression pattern comparable to that of *SOS1*, with additional upregulation in the pericycle. This suggests a broader cellular role for SOS2, potentially including the regulation of Na^+^ loading into the vasculature. Unexpectedly, *SOS3* displayed a widespread downregulation pattern across multiple cell types under salt-stress conditions, diverging from the coordinated upregulation typically expected within the SOS pathway (Supplemental Figures 1B and 1C) (Ji et al., 2013). As noted above, this may reflect a technical artifact associated with protoplasting-induced Ca^2+^ influx, which could lead to elevated expression of calcium-responsive genes under mock conditions (Denyer and Timmermans, 2022). This observation highlights the challenges of using scRNA-seq analysis to detect rapid changes in transcript levels, particularly when calcium sensors are involved. Nevertheless, the expression patterns of other highly cell-type-restricted components are consistent with previous reports (Gámez-Arjona et al., 2024; Ding et al., 2025). *HKT1* exhibited marked downregulation specifically in the pericycle, the sole cell type in which its expression was detectable under these experimental conditions (Supplemental Figure 1C). This observation provides spatial context for the reported SOS3-mediated downregulation of HKT1 protein abundance under severe Na^+^ stress (Gámez-Arjona et al., 2024).

### Single-cell analysis in monocotyledons

**Figure 4:**
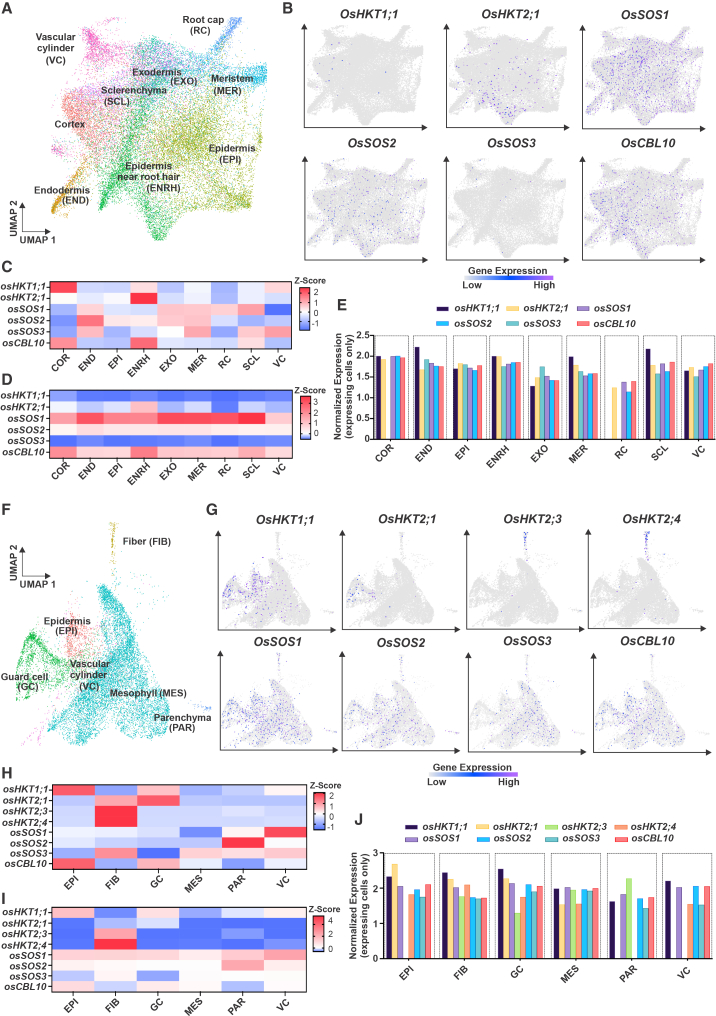
scRNA-seq analysis of Na^+^ homeostasis pathway components in *O. sativa*. **(A)** Two-dimensional UMAP projection and annotation of root clusters derived from transcriptomic profiling of more than 116,564 *O. sativa* single cells across multiple tissues, based on the publicly available dataset GEO: GSE232863 (Wang et al., 2025). **(B)** UMAPs showing cell-type-specific expression patterns of *HKT* genes and components of the SOS signaling pathway in *O. sativa* root cells, grouped into transcriptionally distinct clusters. Genes for which no signal is displayed correspond to cases in which expression was not detected at sufficient levels. **(C)** Heatmap showing the proportion of *O. sativa* root cells expressing each gene within each tissue. Values are row-normalized to emphasize relative expression prevalence across tissues. **(D)** Heatmap showing the proportion of cells expressing each gene across all root tissues, with values globally normalized to allow direct comparison of absolute expression magnitudes. **(E)** Bar plot displaying the average expression level of each gene across root tissues. **(F)** Two-dimensional UMAPs of single-cell transcriptomes from *O. sativa* leaf tissue, revealing transcriptionally differentiated groups corresponding to major leaf regions, as reported by Wang et al. (2025). **(G)** Individual UMAPs displaying the spatial expression patterns of *HKT* genes and components of the SOS signaling pathway across *O. sativa* leaf cell types. Genes not displayed correspond to cases in which expression was either undetectable or below the threshold required for reliable visualization. **(H–J)** Heatmaps **(H and I)** and bar plot **(J)**, corresponding to panels **(C)**, **(D)**, and **(E)**, respectively, showing the proportion of leaf cells expressing each gene across distinct tissue groups.

Monocotyledons typically possess more *HKT* genes than dicotyledons, encoding both class I and class II transporters, in contrast to the single class I *HKT1* gene found in dicotyledons, as previously described (Horie et al., 2001; Huang et al., 2008). scRNA-seq analysis of *O. sativa* root tissues allowed us to map the expression patterns of these two transporter families (Figures 4A–4J) (Wang et al., 2025). *OsHKT1;1* was most highly expressed in the cortex, epidermis near root hairs, and vascular cylinder (Figures 4B and 4C). Although this pattern does not exactly match the pattern observed in *in situ* hybridization experiments, which suggested OsHKT1;1 localization around the stele (Jabnoune et al., 2009), it supports the well-established role of OsHKT1;1 as a Na^+^ uptake barrier. Specifically, OsHKT1;1 retrieves Na^+^ that has traversed the outer root cell layers, thereby preventing its entry into the vascular tissue and subsequent translocation to the shoot (Wang et al., 2015; Campbell et al., 2017). This role differs from that of HKT1 in *A. thaliana*, where AtHKT1;1 resides primarily on the plasma membrane of xylem parenchyma cells (Figure 3) (Davenport et al., 2007; Plett et al., 2010). By contrast, *OsHKT2;1* exhibited the strongest expression in the epidermis, the outermost cell layer of the root (Figures 4B and 4C), consistent with previous *in situ* hybridization experiments (Jabnoune et al., 2009). TThis spatial configuration points to a layered defense system: OsHKT2;1 mediates Na⁺-dependent K^+^ uptake at the root–soil interface, helping to adjust Na^+^/K^+^ balance under K⁺ scarcity or salt exposure; OsHKT1;1 then serves as a deeper barrier, safeguarding internal tissues from excessive Na^+^ accumulation.

scRNA-seq revealed distinct spatial and quantitative specialization of the SOS pathway and CBL sensor genes in *O. sativa* roots. *OsSOS1* displayed broad expression across multiple cell layers, including the exodermis, endodermis, and clusters adjacent to the vasculature, such as the sclerenchyma (Figures 4B and 4C). This pattern suggests a central role for OsSOS1 in regulating Na^+^ movement at internal barriers and controlling ion flux toward vascular tissues, in line with previous reports (El Mahi et al., 2019). *OsSOS2* expression largely coincided with *OsSOS1* expression, reinforcing its function as a key regulator of SOS1 activity. By contrast, *OsSOS3* showed low expression levels throughout the root, with only sporadic expression in some clusters (Figures 4D and 4E). Despite this low transcript abundance, OsSOS3 activity may rely on post-transcriptional regulation or require only low transcript levels for effective signaling during the salt-stress response.

The *CBL* gene family also showed *s*patial and tissue-specific regulation. *OsCBL10* was more highly expressed than *OsSOS3* in *O. sativa* roots, with higher levels in the cortex and epidermis, indicating its importance in Na^+^ homeostasis in the outer root layers (Figures 4B–4E). This constitutive expression implies involvement in functions beyond rapid stress responses, potentially contributing to routine physiological and developmental processes. In contrast to dicotyledons, *OsCBL8* expression was undetectable in roots, implying that its role in Na^+^ management is not constitutive but rather context-dependent and possibly induced only under specific calcium-signaling or stress conditions.

Rice leaf transcriptomic data revealed cell-type-specific expression patterns of Na^+^ homeostasis-related genes, as illustrated in Figures 4F–4J (Wang et al., 2025). *OsHKT2* genes, particularly *OsHKT2;3* and *OsHKT2;4*, reached peak expression in fiber cells (Figures 4G and 4H), where their expression was particularly high. Such localization supports the idea that the Na^+^/K^+^ balance in mechanically supportive cells is likely regulated by this transporter family (Jabnoune et al., 2009; Zhang et al., 2017). By contrast, *OsHKT2;1* was predominantly expressed in guard cells (Figures 4G and 4H), implying a role in directly regulating Na^+^ and possibly K^+^ fluxes linked to stomatal function and guard cell turgor. This process may involve Na^+^–K^+^ co-transport, whereby the simultaneous uptake of both ions facilitates turgor adjustments, with K^+^ retained in the cytosol and Na^+^ sequestered into the vacuole. *OsHKT1;1* expression was detectable in the epidermis, vascular-associated clusters, and guard cells, supporting roles in leaf Na^+^ management that may be related to transpiration-driven and Na^+^ distribution throughout the plant, consistent with previous *in situ* localization studies (Jabnoune et al., 2009).

*OsSOS1* and *OsSOS2* exhibited widespread expression in leaves, which is likely essential for safeguarding photosynthetic tissues during stress (Figures 4G–4I). Their enrichment in parenchyma and vascular cylinder cells also suggests roles in the regulation of long-distance ion transport. While *OsSOS3* transcripts were detected across various cell types, their levels were reduced in guard cells and the epidermis (Figures 4F–4H); conversely, *OsCBL10* was strongly expressed in these regions, consistent with a tissue-specific regulatory mechanism within the CBL–SOS signaling network. *OsCBL10* expression in guard cells implies a role in vacuolar Na^+^ sequestration and stomatal ion homeostasis, potentially mediated through interaction with voltage-gated K^+^ channels, as observed in the CBL10–AKT1 module in *A. thaliana* (Ren et al., 2013). In parallel, *OsSOS3* expression in the vascular cylinder and associated fibers aligns with its established function in stabilizing the SOS1 Na^+^ transporter at the plasma membrane to regulate long-distance Na^+^ transport (Gámez-Arjona et al., 2024). In summary, unlike other stress-related genes with cell-type-restricted expression, such as certain *OsHKT2* isoforms, the widespread expression of *SOS* and *CBL* genes enables systemically coordinated Na^+^ and Ca^2+^ responses under salt stress, promoting robust adaptation across leaf tissues.

### Comparative cell-type-specific expression analysis in monocotyledons and dicotyledons

Based on scRNA-seq data, monocotyledons (*O. sativa*) and dicotyledons (*A. thaliana*) appear to exhibit fundamentally different strategies for regulating ion homeostasis. In roots, *A. thaliana* employs an exclusion-focused strategy with *SOS1*, *SOS2*, and *SOS3* predominantly expressed in the outermost epidermal layers, creating a first line of defense by actively pumping Na^+^ out at the point of entry, while *HKT1* transcription is mainly confined to the pericycle for Na^+^ exclusion from the xylem (Figure 3). In contrast, *O. sativa* utilizes a more internally focused, layered approach, with *OsSOS1* and *OsSOS2* most strongly expressed in the endodermis and internal vascular tissues, suggesting controlled Na^+^ distribution rather than immediate exclusion (Figure 4). This strategy is supported by a highly organized HKT system, in which OsHKT2;1 modulates the early regulation of ion levels in the epidermis, whereas OsHKT1;1 functions as a deeper barrier within the cortex and endodermis. Anatomical differences, such as a wider cortical region in monocotyledons, may underlie their distinct strategy for ionic stress management by facilitating ion redistribution, which may correlate with reduced dependence on SOS1 tonoplast localization in root epidermal cells for ion redistribution (El Mahi et al., 2019; Ramakrishna et al., 2025). The expression pattern of *CBL10* further illustrates these adaptive differences: in *A. thaliana*, *CBL10* is enriched in the root meristem to protect actively growing tissue, whereas in *O. sativa*, *OsCBL10* is highly expressed in the root cortex and epidermis under normal conditions, suggesting that it may act as a frontline regulator.

Monocotyledons employ a wider array of HKT family members from two different subclasses and exhibit more widespread tissue distribution than the largely vasculature-specific *HKT* expression observed in *A. thaliana*, indicating that monocotyledons and dicotyledons have evolved divergent mechanisms for regulating leaf Na^+^ homeostasis. Expression patterns of the SOS pathway genes differed substantially between *O. sativa* and *A. thaliana*. In *O. sativa* leaves, these genes exhibited a notably broader distribution across tissue types (Figures 3 and 4), reflecting a more generalized functional deployment. This pattern indicates that the pathway not only contributes to long-distance Na^+^ transport but also protects photosynthetic tissues. Notably, *OsCBL10* diverged from the *A. thaliana* ortholog by lacking the dominant expression typically observed among other SOS components. Instead, *OsCBL10* showed a cell-specific expression pattern complementary to that of *OsSOS3*, appearing predominantly in cell types where *OsSOS3* expression was low, such as epidermal and guard cells. Despite these differences, both species share a conserved organizational principle: *SOS1* and *SOS2* expression tended to co-localize within the same cell types. Other components, such as *CBL8*, *CBL10*, and *SOS3*, displayed substantial spatial expression heterogeneity across plant taxa. This reflects species-specific evolutionary strategies for ion homeostasis and exemplifies how orthologous proteins can adapt their functions through cell-type-specific expression.

## Subcellular targeting of proteins in plant cells

Regulation of Na^+^ homeostasis encompasses an additional layer of complexity that extends beyond cell-type-specific localization, namely the precise subcellular distribution of ion transporters within individual cells. While previous research has thoroughly elucidated the biochemical mechanisms and functional roles of Na^+^ pathway components, the dynamic processes governing their intracellular trafficking and final subcellular positioning remain incompletely characterized. Recent findings suggest that the subcellular compartmentalization and regulated translocation of SOS components represent critical determinants of effective cellular responses to salt stress (Gámez-Arjona et al., 2024; Salazar et al., 2024; Liu et al., 2025a; Ramakrishna et al., 2025). Key post-translational modifications (PTMs) govern SOS3 subcellular localization and function. N-myristoylation of glycine-2 provides the plasma membrane anchor required for salt-stress activation (Villalta et al., 2021), facilitating SOS2 recruitment through a specialized FISL/NAF interaction motif (Quintero et al., 2002; Sánchez-Barrena et al., 2007). S-acylation of cysteine-3 directs a subset of SOS3 proteins to the nucleus, revealing regulatory functions beyond membrane signaling. These functions include the modulation of flowering time under salt stress via interaction with GI to modulate *CONSTANS* expression (Park et al., 2023). Additionally, under salt-stress conditions, SOS3 facilitates the recruitment of SOS1 to the plasma membrane, enhancing Na^+^ tolerance (Gámez-Arjona et al., 2024). SORTING NEXIN 1 is also required for salt-stress tolerance by regulating endosomal trafficking of SOS1 in *A. thaliana* (Song et al., 2024), which may be related to late endosomal and tonoplast localization of SOS1 (Ramakrishna et al., 2025). Intriguingly, SOS2 has been detected in association with endosomal markers, indicating its direct participation in trafficking events beyond its canonical cytosolic signaling role (Liu et al., 2025a). The subcellular regulation of SOS components suggests that their spatial arrangement within cellular compartments and dynamic redistribution in response to environmental signals are as critical as tissue-level expression patterns in determining plant salt tolerance.

An important aspect of ion transport is the precise distribution of key components within a given subcellular compartment. For example, proteins at the plasma membrane may concentrate in defined domains, influencing their activity, interactions, and the establishment of cellular polarity that directs ion flow. Under salt stress, Na^+^ homeostasis in roots depends on the coordinated vectorial activity of SOS1 and HKT1 to control long-distance Na^+^ transport to the shoot (Gámez-Arjona et al., 2024). However, the molecular mechanisms underlying the directional placement of Na^+^ transporters remain largely unresolved, especially when compared with well-characterized systems governing other transport processes. In well-established cases, transporter polarity is tightly controlled by cellular machinery, including vesicular trafficking, which targets proteins to specific membrane domains, and PTMs such as phosphorylation and ubiquitination, which act as molecular switches. For instance, the polarity of PIN auxin transporters is controlled by phosphorylation (Rademacher and Offringa, 2012; Zhang et al., 2023), BOR1 polarity is maintained via AP2-dependent endocytosis under low-boron conditions (Yoshinari et al., 2019), and the polar localization of OsLsi1, essential for silicon uptake in rice, depends on positively charged residues at its C-terminus (Konishi et al., 2023). Interestingly, Ca^2+^ signals are required to establish the polarity of auxin transport (Li et al., 2019a), and given their central role in Na^+^ homeostasis, they may similarly shape the subcellular trafficking of Na^+^ transporters. In summary, plant cells integrate metabolic and environmental signals to regulate subcellular distribution and ion transporter polarity, thereby controlling cytosolic ion concentrations. Although operating at different scales, this cellular-level regulation may reflect analogous Na^+^ partitioning strategies observed at the whole-plant level, suggesting conserved principles across levels of biological organization.

## Concluding remarks and future perspectives

### Fundamental cellular and physiological mechanisms

Critical knowledge gaps persist in understanding the mechanisms of Na^+^/K^+^ balance underlying plant salt tolerance. Although potential Na^+^ stress sensors, such as glycosyl inositol phosphorylceramide sphingolipids, have been proposed (Jiang et al., 2019), their integration into known signal transduction networks has yet to be elucidated. An important direction for future research lies in dissecting the spatiotemporal trafficking of SOS1 across various organelles, including the plasma membrane, endomembrane system, vacuole, and endoplasmic reticulum. A related unresolved question concerns the identity of SOS1-transporting vesicles and the trafficking machinery that controls the targeted delivery and recycling of SOS1. Addressing this question will require clarifying how salt-induced changes in membrane lipid composition influence SOS protein activity, stability, and mobility. Membrane lipid environments influence membrane organization and modulate the activity of signaling proteins, including kinases, phosphatases, and ubiquitin ligases (Morales-Cedillo et al., 2015). Integrating lipidomics with analyses of phosphorylation, ubiquitination, and other PTMs may reveal how plant cells dynamically establish and maintain the spatial patterns and polarity of Na^+^ transporters, with direct consequences for ion homeostasis, nutrient uptake efficiency, detoxification, and root architectural responses. Furthermore, this approach would facilitate the exploration of functional crosstalk between Na^+^ transporters and other essential membrane-associated systems, including PIN-mediated auxin efflux and BRI1-dependent brassinosteroid signaling, both of which are central to root development and salt-stress adaptation (Yang et al., 2022; Blanco-Touriñán et al., 2024). At the whole-plant level, these molecular mechanisms must be understood in the context of strong tissue- and cell-type specificity. Examining transporter activity in discrete root regions may reveal different strategies that are otherwise masked in bulk analyses. In addition, substantial uncertainty remains regarding the relative contributions of the two major phases of salt stress, namely the early osmotic shock and the later ionic toxicity caused by intracellular Na^+^ accumulation (Munns and Tester, 2008). High-precision phenotyping efforts therefore need to distinguish between meristematic and mature tissues, which differ significantly in physiology, stress sensitivity, and protective barriers. The root meristem, for example, lacks a Casparian strip yet experiences rapid cell division and intense signal flux, making it a uniquely valuable target for studying early salt-stress detection and adaptation mechanisms (Alonso Baez et al., 2026). Finally, understanding whole-plant responses requires integrating these molecular insights with the functional status of chloroplasts (Müller et al., 2014) and mitochondria, both of which deploy specialized protective mechanisms against Na^+^ toxicity. How these organellar processes interface with systemic signaling and long-distance Na^+^ transport remains poorly understood and represents an important frontier for future research.

### Technical approaches

Advances in salt tolerance research increasingly rely on high-resolution, multi-dimensional analytical techniques. Among the most powerful emerging approaches is cryo-nanoscale secondary ion mass spectrometry (cryo-nanoSIMS), a technique that provides subcellular elemental mapping with sensitivity down to the parts-per-million range. This level of resolution has been used to challenge traditional models by revealing novel roles for extensively studied proteins such as SOS1 (Ramakrishna et al., 2025). This approach can be applied to additional transporters to uncover previously overlooked subcellular activities. Single-cell profiling can also help identify cell-type- and tissue-specific regulatory programs, enabling targeted expression of engineered transporters in the most relevant plant compartments to enhance ion mobilization and promote plant fitness. Dynamic molecular biosensors complement these static imaging approaches. Genetically encoded indicators such as GINKO2 for K^+^ (Wu et al., 2022) and near-infrared-shifted Na^+^ probes (Ma et al., 2023) allow simultaneous, real-time monitoring of ionic ratios with minimal spectral overlap. The dual Na^+^/K^+^ in planta microneedle sensor provides rapid (< 5 s) *in vivo* measurements of vascular ion fluxes (Wang et al., 2024b). This capacity enables the detection of early ionic waves and growth-defense trade-offs that occur before visible symptoms emerge. Additional tools, including PAleon for phosphatidic acid (Li et al., 2019b), ABA sensors such as ABAleon and ABACUS2 (Waadt et al., 2014; Rowe et al., 2023), and Ca^2+^ (Liese et al., 2023) and reactive oxygen species reporters (Akter et al., 2021), make it possible to track the coordinated signaling that drives early stress responses. Choosing between nuclear-localized and cytosolic versions of these sensors will remain an important experimental consideration. At the regulatory level, targeted mass spectrometry, proximity labeling using TurboID, and PTM mapping provide routes to uncover new modulators of SOS proteins and to resolve the signatures of osmotic versus ionic stress signaling. Additionally, mapping the influence of transposable elements on transcriptional networks offers a novel approach to dissecting the regulatory architecture governing global Na^+^ transport and cellular homeostasis (Tossolini et al., 2025). Leveraging these structural variations to understand how plants orchestrate ion partitioning may uncover unexploited genetic targets for engineering salt-resilient crops. This integrative approach, which combines spatial partitioning through cryo-nanoSIMS, temporal dynamics via biosensors, and mechanistic insights from PTM mapping, is essential for developing the next generation of salt-tolerant crops.

### Practical pathways and applications

Harnessing natural variation in *SOS* and *HKT* genes and their regulators across species, and combining this knowledge with gene editing, marker-assisted breeding, and the study of gene duplication and neofunctionalization in halophytes, offers promising opportunities for discovering novel salt tolerance strategies and engineering resilient crops (Zhou et al., 2022; Salazar et al., 2024). Cross-species comparative analysis remains a powerful tool for identifying mechanistic innovations that cannot be inferred from *Arabidopsis* alone. To maximize the efficacy of these genetic interventions, insights gained from single-cell and spatial transcriptomics provide a high-resolution blueprint for cell-type-specific engineering. By identifying the specific cellular niches where salt-responsive genes are naturally active, researchers can use targeted promoters, such as SUC2 for phloem companion cells, APL for phloem, WER for root atrichoblasts, RbcS for green parenchyma, and GC1 for guard cells, to drive expression of transporters and tolerance genes with high spatial precision. This multi-scale approach enables the transition from whole-plant overexpression to nuanced, “smart” interventions. Microbiome-based interventions represent an expanding frontier, as microbial interactions influence Na^+^ uptake, osmotic adjustment, and oxidative stress mitigation (González Ortega-Villaizán et al., 2026). Finally, laboratory insights must be validated in heterogeneous agricultural field environments, in which multiple abiotic stresses overlap and soil microbiomes strongly shape plant responses. Integrating an ecophysiological perspective will be essential to ensure the successful translation of mechanistic discoveries into robust agricultural outcomes.

## Funding

This work was supported by grants from the 10.13039/501100011033Spanish State Research Agency (MICIU/AEI/10.13039/501100011033, Spain): PID2023-149567NA-I00 to F.M.G.-A., PID2022-140705OB-I00 to F.J.Q., and PID2024-160398NB-I00 to J.M.P. F.M.G.-A. was supported by a Ramón y Cajal Fellowship (RYC2022-035325-I), and A.G.D.l.R. was supported by a Juan de la Cierva Fellowship (JDC23-050648-I), both from AEI-MCIN (Spain). All grants and fellowships were co- funded by the 10.13039/501100008530European Regional Development Fund.

## Acknowledgments

We are grateful to our colleagues at the University of Seville and CSIC for their valuable input and feedback. We sincerely apologize to any researchers whose contributions we may have inadvertently overlooked in this review. No conflict of interest declared.

## Author contributions

F.M.G.-A. conceptualized and designed the study. V.J.F.-R. performed the single-cell analysis, and A.G.D.l.R. contributed to data analysis and interpretation. V.J.F.-R. and A.G.D.l.R. prepared the figures and visualizations. F.M.G.-A., V.J.F.-R., A.G.D.l.R., F.J.Q., and J.M.P. drafted and revised the manuscript and figures. All authors approved the final manuscript for submission.

## Supplemental information

Document S1. Supplemental Figure 1 and supplemental data

Document S2. Article plus supplemental information
