# Supplementary material for: The architecture of salt tolerance: A multi-scale view of sodium transport in plants
Source: Plant Commun. 2026 May 12;7(6):101888. doi: 10.1016/j.xplc.2026.101888 (PMC13261650; doi:10.1016/j.xplc.2026.101888)
Supplement: Document S1. Supplemental Figure 1 and supplemental data [file mmc1.pdf]

Plant Communications, Volume *7*

Supplemental information

The architecture of salt tolerance: A multi-scale view of sodium transport in plants

Víctor J. Fernández-Ramírez, Alfonso G. De la Rubia, Jose M. Pardo, Francisco J. Quintero, and Francisco M. Gámez-Arjona

Supplemental information

The architecture of salt tolerance: A multi-scale view of sodium transport in plants

Víctor J. Fernández-Ramírez*, Alfonso G. De la Rubia*, Jose M. Pardo, Francisco J. Quintero, and Francisco M. Gámez-Arjona.

Institute of Plant Biochemistry and Photosynthesis, Spanish National Research Council (CSIC) – University of Seville, Seville 41092, Spain.

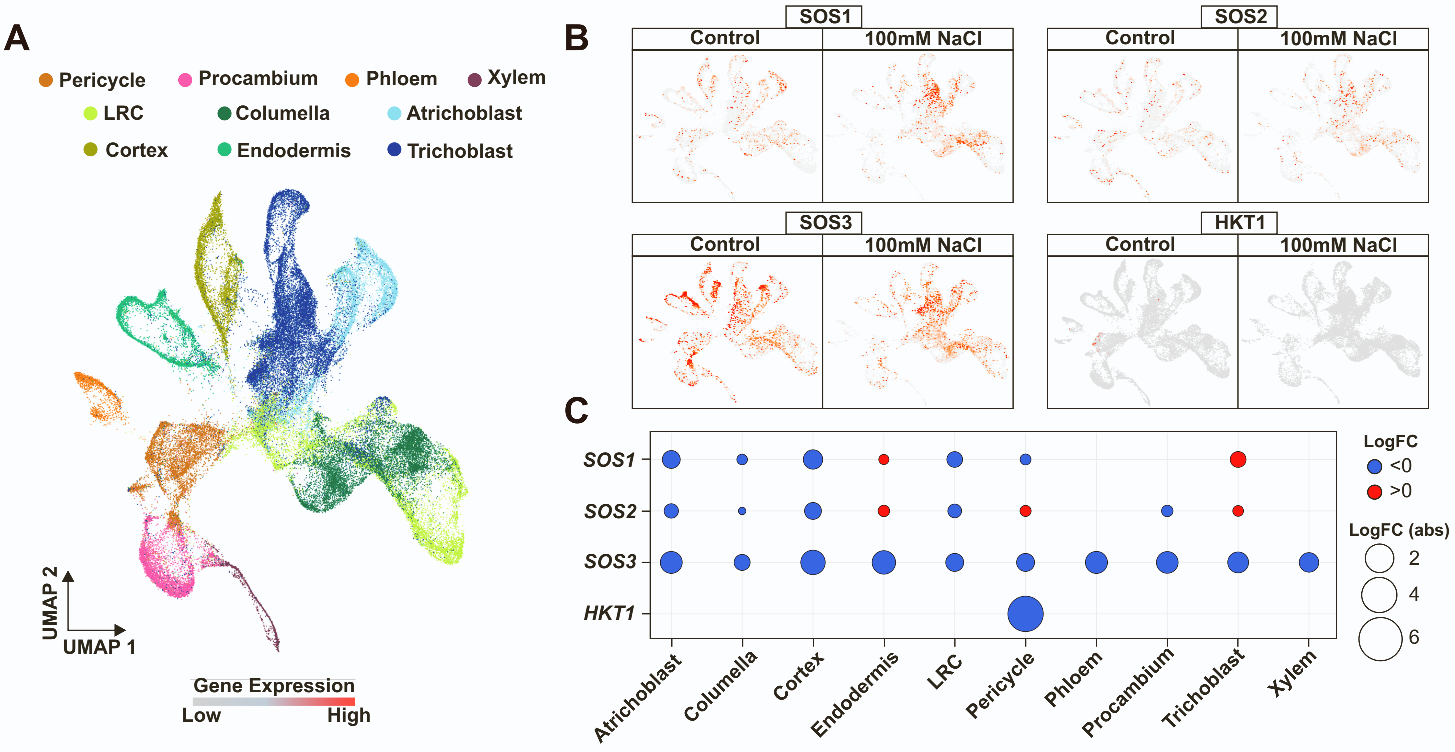

Supplementary Figure 1. Salt-induced transcriptional reprogramming of SOS pathway components in *A. thaliana* root cells under salt stress. (A) UMAP embedding of single-cell transcriptomes from *A. thaliana* roots, derived from a high-resolution atlas integrating transcriptomic profiles across multiple stress conditions (Wang et al., 2024b). Cells were computationally clustered based on transcriptional similarity, enabling the delineation of discrete root cell populations. (B) Comparative expression patterns of four core SOS pathway genes—*SOS1, SOS2, SOS3, and HKT1*—across cell clusters under control and salt stress conditions (100 mM NaCl). (C) Bubble plot summarizing the log fold change (logFC) in gene expression (NaCl vs. control) across major root tissues. Bubble size reflects the absolute magnitude of logFC while color indicates directionality of expression change (red: upregulated; blue: downregulated). Absence of a bubble denotes no detectable differential expression (logFC = 0).

Supplementary File 1

Publicly available single-cell RNA sequencing (scRNA-seq) datasets from *Arabidopsis thaliana* (A. thaliana) and *Oryza sativa* (O. sativa) were examined, focusing on root and leaf tissues. *A. thaliana* data were obtained from The Plant scRNA-Seq Browser (Denyer et al., 2019; Kim et al., 2021; Ma et al., 2020), corresponding to GEO accession numbers GSE123818, GSE123013, GSE161482, and GSE152766. The *A. thaliana* salt stress dataset was retrieved from Wang et al. (2024b); as this dataset is not yet publicly released under GEO accession GSE268881, data were accessed via the authors' interactive portal (https://plantbiology.shinyapps.io/athatlas/). The *O. sativa* dataset was retrieved from Wang et al. (2025), available under GEO accession number GSE232863.

For *A. thaliana* datasets we utilized the original data provided by the authors, which included log-normalized count matrices and curated metadata. These data were used to compute gene-level metrics, including the proportion of cells expressing each gene and the average expression level within defined tissue subsets. To support interpretation and maintain consistency with published annotations, we consulted the authors' interactive browser (https://www.zmbp-resources.uni-tuebingen.de/timmermans/plant-single-cell-browser-root-atlas/). The cell-type annotation maps and gene expression plots presented were adapted directly from this resource and selected to illustrate transcriptional features relevant to our comparative framework. To preserve local structure and highlight subtle transcriptional differences across root and leaf cell populations, t-distributed stochastic neighbor embedding (t-SNE) plots were generated and used as the basis for visualizing cell-type distributions. The analytical steps described below are those implemented in the original studies and are summarized here for completeness and transparency.

For the root tissue dataset, raw reads were aligned to the Arabidopsis TAIR10 reference genome using Cell Ranger (v2.0.2), with the STAR aligner employed for mapping and gene expression matrix generation. Valid cell barcodes were defined dynamically based on a UMI distribution threshold (cell read count > 5% of the 99th percentile of 7,000 cells). The resulting dataset featured a median of 4,276 genes and 14,758 UMIs per cell. Dimensionality reduction and clustering were conducted using Seurat (v2.3.4), selecting highly variable genes based on a dispersion z-score cutoff >1. Principal

Component Analysis (PCA) was performed using 50 principal components, followed by graph-based clustering at a resolution of 0.8. Cluster robustness was further validated using a random forest classifier, merging clusters only when the out-of-bag error (OOBE) exceeded 10%. For the leaf tissue dataset, raw reads were aligned to the Arabidopsis reference genome (Araport 11) using Cell Ranger (v3.0.2). Data processing was performed using Seurat (v3.1.0) with normalization and variance stabilization conducted via the SCTransform method, which explicitly regressed out mitochondrial transcript abundance. This dataset comprised a median of 3,342 genes and 27,159 UMIs per cell. Clustering utilized the top 50 PCs at a resolution of 0.8, while visualization was generated via Uniform Manifold Approximation and Projection (UMAP) using 10 principal components, 30 neighboring points, and a minimum distance of 0.1. Finally, for both datasets, cell-type identities were assigned using unbiased marker-gene selection based on the Seurat likelihood-ratio test (Bimod). To ensure high-confidence assignments, marker genes were defined using stringent statistical thresholds: alongside an adjusted p-value < 0.01 and an average natural log fold change ≥ 0.25, genes were required to be detected in > 10% of cells within the target cluster (PCT1) and in < 10% of background cells (PCT2).

For the *A. thaliana* salt stress dataset, cell-type specific transcriptomic features and published annotations were explored directly through the authors' interactive data portal. Given that the raw datasets were under restricted access at the time of this review, this platform served as the primary benchmark for interpreting the root atlas and ensuring consistency with the established cell-type maps. The analytical workflow implemented in the original study is outlined hereafter. Raw data were processed using Cell Ranger (v5.0.1) and aligned to the TAIR10 reference genome. Doublet detection was performed using DoubletFinder (v3), applying a 7.5% expected doublet rate. Cells were further filtered to include only those with 400 to 10,000 expressed genes and < 5% mitochondrial reads. Additionally, genes expressed in fewer than 3 cells were excluded from the analysis. Data integration and clustering were conducted in Seurat (v4.0.5), where 2,000 highly variable genes were identified via variance-stabilizing transformation. Dimensionality reduction was performed using PCA followed by UMAP visualization with 50 PCs. Cell-type identities were assigned through a multi-method

consensus approach, combining correlation-based, marker-based (using SEMITONES), and integration-based annotations from reference atlases. While this workflow ensures robust cell mapping, it is important to note that the protoplasting process can induce rapid Ca^{2+} influx. This technical limitation may affect the detected levels of calcium-sensitive transcripts, such as *SOS3*, and should be considered when interpreting the expression patterns of signaling components, especially given the central role of Ca^{2+} homeostasis in the plant's response to salt stress.

For *O. sativa*, the dataset was similarly retrieved from the original publication, including pre-processed expression matrices and curated metadata provided by the authors. Following acquisition, we performed tissue-specific filtering to isolate root and leaf cell populations, ensuring consistency with the comparative framework established for *A. thaliana* previously. *O. sativa* data were visualized using UMAP plots, which offers scalability and global structure preservation, particularly suited to the larger and more heterogeneous *O. sativa* dataset. To characterize gene activity across tissues, we filtered cells based on annotated tissue identity and computed two key metrics: the proportion of cells expressing each gene, and the average expression level of each gene within each tissue subset. This approach enabled high-resolution comparative transcriptomic profiling, while maintaining fidelity to the biological and technical assumptions embedded in each dataset. The analytical workflow applied in the original study is summarized here to ensure methodological transparency. Briefly, raw data were processed using CellRanger (v8.0.0) with the Rice MSU 7.0 genome as reference. Low-quality cells (<500 genes or <1,000 UMIs) and doublets were removed using DoubletFinder (v2.0.3), and data were normalized using the NormalizeData and ScaleData functions in Seurat (v5.1.0). The top 2,000 highly variable genes were selected, PCA was performed using 50 principal components, and batch effects across organs were corrected using Harmony (v0.1.0). Clustering was conducted using a KNN-based approach at a resolution of 0.2, and visualization was performed using UMAP based on the first 20 dimensions. Cell-type identities were assigned based on differentially expressed marker genes identified using the Wilcoxon rank-sum test, with thresholds of adjusted p-value < 0.05, $|\log2 \text{ fold change}| > 0.25$, and expression in > 25% of cells within the target cluster.

Downstream computational analyses and visualizations were performed in R (v4.5.1) using the Seurat (v5.3.0) framework, providing a consistent methodological approach for all publicly available objects. Detailed scripts used for this study are available from the corresponding author upon reasonable request.
